# Loss of Atrx Sensitizes Cells to DNA Damaging Agents through p53-Mediated Death Pathways

**DOI:** 10.1371/journal.pone.0052167

**Published:** 2012-12-17

**Authors:** Damiano Conte, Michael Huh, Emma Goodall, Marilyne Delorme, Robin J. Parks, David J. Picketts

**Affiliations:** 1 Regenerative Medicine Program, Ottawa Hospital Research Institute, Ottawa, Ontario, Canada; 2 Department of Cellular and Molecular Medicine, University of Ottawa, Ottawa, Ontario, Canada; 3 Department of Biochemistry Microbiology and Immunology, University of Ottawa, Ontario, Canada; 4 Department of Medicine, University of Ottawa, Ottawa, Ontario, Canada; University of Montréal and Hôpital Maisonneuve-Rosemont, Canada

## Abstract

Prevalent cell death in forebrain- and Sertoli cell-specific *Atrx* knockout mice suggest that Atrx is important for cell survival. However, conditional ablation in other tissues is not associated with increased death indicating that diverse cell types respond differently to the loss of this chromatin remodeling protein. Here, primary macrophages isolated from *Atrx* ^f/f^ mice were infected with adenovirus expressing Cre recombinase or β-galactosidase, and assayed for cell survival under different experimental conditions. Macrophages survive without Atrx but undergo rapid apoptosis upon lipopolysaccharide (LPS) activation suggesting that chromatin reorganization in response to external stimuli is compromised. Using this system we next tested the effect of different apoptotic stimuli on cell survival. We observed that survival of Atrx-null cells were similar to wild type cells in response to serum withdrawal, anti-Fas antibody, C2 ceramide or dexamethasone treatment but were more sensitive to 5-fluorouracil (5-FU). Cell survival could be rescued by re-introducing Atrx or by removal of p53 demonstrating the cell autonomous nature of the effect and its p53-dependence. Finally, we demonstrate that multiple primary cell types (myoblasts, embryonic fibroblasts and neurospheres) were sensitive to 5-FU, cisplatin, and UV light treatment. Together, our results suggest that cells lacking *Atrx* are more sensitive to DNA damaging agents and that this may result in enhanced death during development when cells are at their proliferative peak. Moreover, it identifies potential treatment options for cancers associated with ATRX mutations, including glioblastoma and pancreatic neuroendocrine tumors.

## Introduction

Constitutional mutations in the *ATRX* gene cause a rare form of X-linked intellectual disability, namely the α-thalassemia mental retardation syndrome (ATR-X; OMIM# 30032) [1]. To date, more than 200 affected individuals have been identified in 182 families worldwide, and ATR-X is estimated to affect 1-9/1,000,000 births [2], [3]. Individuals with ATR-X syndrome are characterized by severe intellectual disabilities, alpha thalassemia, urogenital dysfunction, skeletal abnormalities, and neonatal muscular hypotonia [2], [3]. Most disease causing mutations are missense changes located within two highly conserved regions, an N-terminal ADD domain (an atypical PHD domain common to ATRX, DNMT3 and DNMT3L) and a C-terminal ATPase/helicase motif shared by the many Swi2/Snf2-like chromatin remodeling proteins.

These two domains also define the known biochemical properties and functions of the ATRX protein. The ADD domain forms a pocket for binding H3K4me^0^/H3K9me^3^ histone tails that are enriched in heterochromatin and mediate ATRX localization to pericentromeric heterochromatin [4], [5], [6]. Heterochromatin binding is also facilitated by interactions with HP1α and MeCP2 [7], [8], [9], [10]. The ATPase domain is most similar to RAD54 and, in a complex with the death domain-associated (Daxx) protein, is necessary for DNA translocase activity and to remodel mononucleosomes [11], [12]. Additionally, ATRX is known to associate with promyelocytic leukemia nuclear bodies (PML-NBs) where it also co-localizes with Daxx [11], [12]. Furthermore, Daxx-ATRX complexes are necessary for the deposition of the histone variant H3.3 at pericentromeric and telomeric heterochromatin [13], [14], [15]. Despite these advances in our understanding of ATRX biochemical function it is not clear how these activities contribute to disease pathology.

Patient mutations appear to be functional hypomorphs that attenuate ATPase activity and affect the localization of the protein to PML-NBs and heterochromatin [11], [16]. Other studies demonstrated that methylation at rDNA and Y-chromosome specific repeats are altered in patient cell lines [17]. Recent studies showed that ATRX binds to G4 quadruplexes *in vitro* and that reduced α-globin expression in ATR-X patients may arise from unfettered formation of G4 structures within a variable tandem repeat upstream of the globin locus [18]. Inactivation of *Atrx* in mice has indicated a survival requirement for Atrx within the early embryo, for neuronal survival during corticogenesis and for Sertoli cell survival in the developing gonad [19], [20], [21]. Cell death could be partially rescued in the forebrain by removal of p53 suggesting that Atrx could be important for maintaining genomic stability [22]. However, Atrx ablation in the retina and in bone is not associated with extensive apoptosis suggesting that the function of Atrx in cell survival may be more complex [23], [24]. In this regard, several other studies have implied that stress signaling, cell-cell signaling or Daxx-mediated pathways are important survival mechanisms for Atrx-deficient cells [24], [25], [26], [27]. Further complicating a role for ATRX in cell survival is the finding that somatic mutations have been reported in several types of cancer [28], [29], [30].

In this study, we developed primary cell cultures from *Atrx^f/f^* mice and infected them with Adenovirus expressing either Cre or LacZ to investigate how ATRX regulates cell survival in an otherwise genetically identical background. Using this approach, different cell types were tested for their sensitivity to various death-inducing stimuli. We observed a general sensitivity to DNA damaging agents that could be rescued by removing p53, suggesting that Atrx plays a role in maintaining DNA integrity and preventing activation of p53-mediated apoptosis.

## Results

### Macrophages Survive in the Absence of Atrx but Undergo Rapid Apoptosis Upon LPS Stimulation

Studies in the forebrain have suggested that Atrx is critical for cell survival, acting through a p53-dependent pathway [20], [22]. Other studies have implied that stress signaling, cell-cell signaling or Daxx-mediated pathways are important survival mechanisms for Atrx-deficient cells [24], [25], [26], [27]. Defining a precise mechanism of Atrx function is limited by the observations that Atrx-null ES cells display a growth disadvantage while primary cell lines derived from transgenic mice have proven difficult to establish due to the early lethality of the embryos [21]. To overcome these difficulties and explore the mechanisms contributing to cell death in *Atrx* mutant mice we utilized cells harvested from *Atrx* ^f/f^ mice followed by infection with adenoviruses expressing either β-galactosidase (AdLacZ) as a control, or Cre-recombinase (AdCre) to inactivate the *Atrx* gene. Macrophages were chosen initially as they are easily harvested from peripheral blood, amenable to experimental analyses and responsive to differentiation conditions.

**Figure 1:**
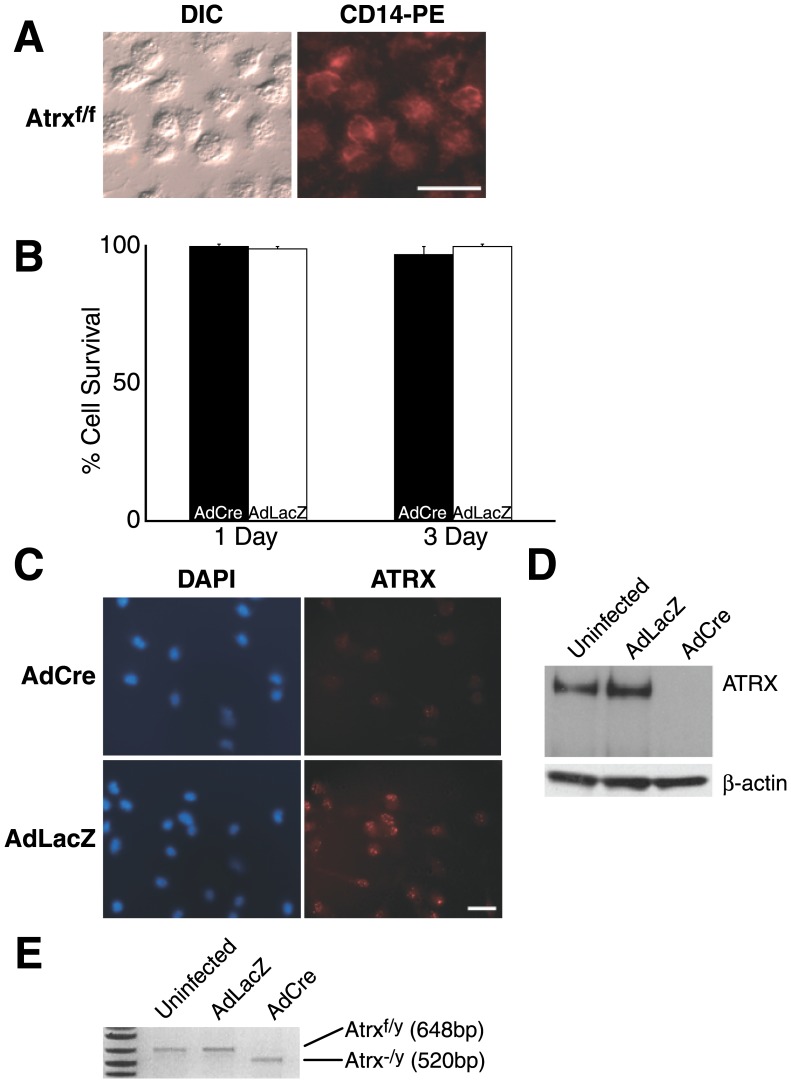
Characterization of primary macrophage experimental cell system. (A) Peritoneum-derived macrophage cell preparations isolated from *Atrx* ^f/f^ mice (left panel) were tested for their purity by staining with the monocyte/macrophage-specific marker CD14 (right panel). DIC - differential interference contrast image. Scale bar: 25 mM. (B) Macrophages infected with AdCre (black bars) or AdLacZ (white bars) were monitored for survival at 1 and 3 days post-infection. (C) Cells immunostained for Atrx 3 days following AdCre or AdLacZ infection. Cells were counterstained with DAPI. Scale bar: 50 mM. (D) Representative immunoblot for Atrx or β-actin 3 days following AdLacZ, AdCre or no treatment. (E) PCR amplification encompassing exon 18, the sequence targeted for deletion. Infection with AdCre but not AdLacZ results in efficient excision of exon 18.

To assess the purity of our isolation procedure we stained the cells for the membrane glycoprotein CD14 (Fig. 1A), a marker expressed strongly on the surface of monocytes and macrophages [31]. We observed positive staining in all cells confirming that our starting population was mononuclear leukocytes. Cultures from individual isolates were divided with one half infected with AdLacZ and the other half infected with AdCre and then examined for cell survival. We observed no differences between AdLacZ or AdCre treated cells at 1 and 3 days post infection (Fig. 1B). Cell staining and immunoblot for Atrx three days post-infection confirmed that the protein was absent (Fig. 1C, D) and PCR of genomic DNA demonstrated that the efficiency of Cre excision approached 100% in these cells (Fig. 1E).

**Figure 2:**
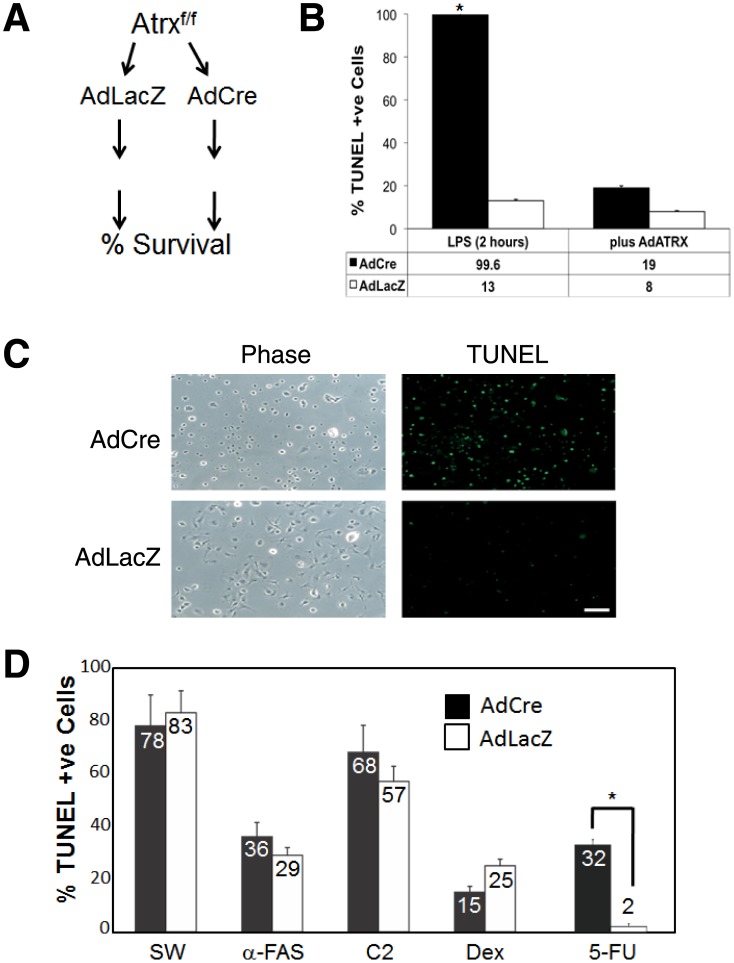
Atrx-null macrophages are sensitive to LPS and 5-fluorouracil treatment. (A) Schematic diagram outlining experimental procedure. Isolated macrophages were split with half the culture infected by Adenovirus expressing β-galactosidase (Ad-LacZ) and the other half infected with Adenovirus expressing Cre recombinase (Ad-Cre). Following Ad infection, cells were treated with different apoptotic inducing stimuli (eg. C2-ceramide) and cell survival was assessed. (B) AdCre cells but not AdLacZ cells were highly sensitive to treatment with LPS (20 ng/ml) for 2 hours. Addition of AdAtrx rescued the viability of these cells as measured by TUNEL assay. (C) Representative images of TUNEL stained cells from AdCre and AdLacZ infected cells 2 hours after LPS treatment. Scale bar: 100 mM. (D) Plot of TUNEL positive cells of AdCre or AdLacZ infected macrophages following serum withdrawal (SW), or treatment with anti-FAS antibody (α-FAS), C2-Ceramide (C2), dexamethasone (Dex), or 5-fluorouracil (5-FU). Numbers represent mean values (n = 4) of TUNEL positive cells. Error bars represent SEM. Statistically significant changes (p<0.05) are denoted by an asterisk.

Since the AdCre and AdLacZ infected cells survived equally well we reasoned that they could be a good model system to test the response of cells to different treatment regimes (Fig. 2A). To test the model system, we treated the AdLacZ or AdCre infected cells with bacterial lipopolysaccharide (LPS). LPS activates macrophages by binding to the CD14 receptor and activating signaling cascades that culminates in a strong transcriptional response, largely characterized by the expression and secretion of numerous cytokines indicative of the immune response [31], [32]. Surprisingly, AdCre treated cells underwent rapid and almost complete apoptosis (99.6% TUNEL positive) within 2 hours after treatment. By comparison, only 13% of the AdLacZ cells treated with LPS (20 ng/ml, 2 hours) were TUNEL positive (Fig. 2B, C). The cell death response was a direct result of the loss of *Atrx* expression because re-introducing *Atrx* using an Adenovirus expressing Atrx (AdAtrx) completely rescued the survival of the macrophages (Fig. 2B). We conclude from this experiment that macrophages can survive in the absence of *Atrx* but are severely compromised in their ability to invoke a response to an external signal such as LPS.

### *Atrx* Deficient Macrophages are Sensitive to 5-fluorouracil Treatment but not Other Apoptotic-inducing Stimuli

**Figure 3:**
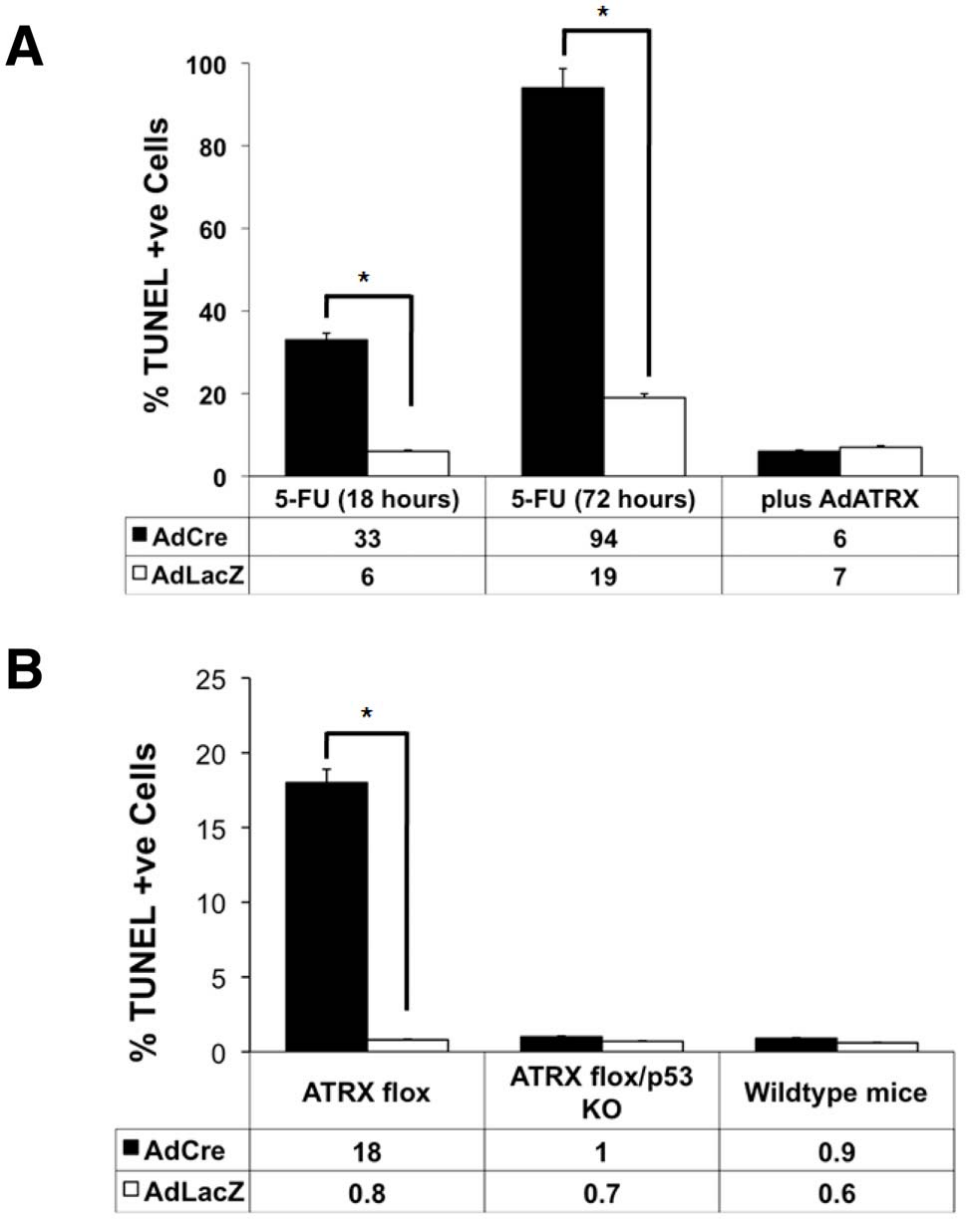
Sensitivity to 5-FU can be rescued by infection with Adenovirus-expressing Atrx. (A) AdCre cells (black bars) were sensitive to 5-FU treatment compared to AdLacZ cells (white bars) at 18 and 72 hours. Co-infection with AdAtrx (plus AdATRX) could rescue the sensitivity to 5-FU treatment (0.5 mM, 18 hr). (B) Similarly, the sensitivity to 5-FU could be rescued by ablation of p53. Cells were isolated from *Atrx* flox, *Atrx* flox/*p53* KO, or WT mice and infected with AdCre (black bars) or AdLacZ (white bars) prior to 5-FU treatment (0.5 mM, 18 hr). The graph plots the percentage of TUNEL positive cells for each experimental condition. Values correspond to the Mean ± SEM (n = 3). Statistically significant changes (p<0.05) are denoted by an asterisk.

Given the sensitivity of the *Atrx*-null cells to LPS, we reasoned that our macrophage model system could be extended to test their sensitivity to different apoptosis-inducing stimuli and allow us to further explore the mechanisms through which Atrx regulates cell survival. We exposed AdLacZ and AdCre infected cells to serum withdrawal (SW; 18 hours), C2-ceramide (C2; 40 mg/ml, 24 hours), dexamethasone (Dex; 100 nM, 12 hours), anti-FAS antibody (α-FAS; 20 mg/ml, 4 hours), or 5-fluorouracil (5-FU; 0.5 mM for 24 hours) and monitored the fraction of TUNEL positive cells as a measure of apoptosis. As shown in Fig. 2D, we observed no significant difference in cell survival under every condition except for treatment with 5-FU where AdCre cells demonstrated far greater sensitivity to the pyrimidine analog (32±2% vs. 2±1%; n = 3; p<5.33×10^−6^). We repeated the experiment looking at both earlier and later timepoints. After 18 hours of exposure to 5-FU, we observed a similar result to treatment for 24 hours with 33±2.6% of the AdCre population TUNEL positive compared to only 6±3% (n = 3; p<0.0003) of the AdLacZ treated population (Fig. 3A). Moreover, when these cells were exposed to 5-FU for 72 hours the *Atrx*-null population was almost completely TUNEL positive (94±2%) whereas the wild type cells were moderately affected (19±2% TUNEL positive; n = 3; p<1.34×10^−6^). Similar to LPS treatment, we could rescue cell survival of the 5-FU treated cells by re-introducing Atrx via co-infection with AdAtrx (6±1.3% AdCre+Atrx; 7±2.6% AdLacZ+Atrx; n = 3; p<0.71; Fig. 3A; Fig. S1).

Since 5-FU is a nucleotide analog and acts primarily through the intrinsic pathway we asked whether we could also rescue the sensitivity of *Atrx*-null cells by removing p53. Previous work has demonstrated that the enhanced death of neural progenitors observed in forebrain-specific conditional *Atrx* KO mice was p53-dependent [22]. As such, we tested the p53-dependence in our macrophage model by crossing the *p53*-null mice onto our *Atrx* ^f/f^ line. The loss of p53 rescued the lethality of 5-FU treatment as we observed no differences in the proportion of TUNEL positive cells in the p53^−/−^Atrx^f/f^ macrophages infected with AdCre compared to those infected with AdLacZ (Fig. 3B). Taken together these results suggest that *Atrx*-null cells are sensitive to apoptotic stimuli that function through the intrinsic pathway via p53-dependent pathways.

### Multiple Cell Types are Sensitive to 5-FU Treatment

**Figure 4:**
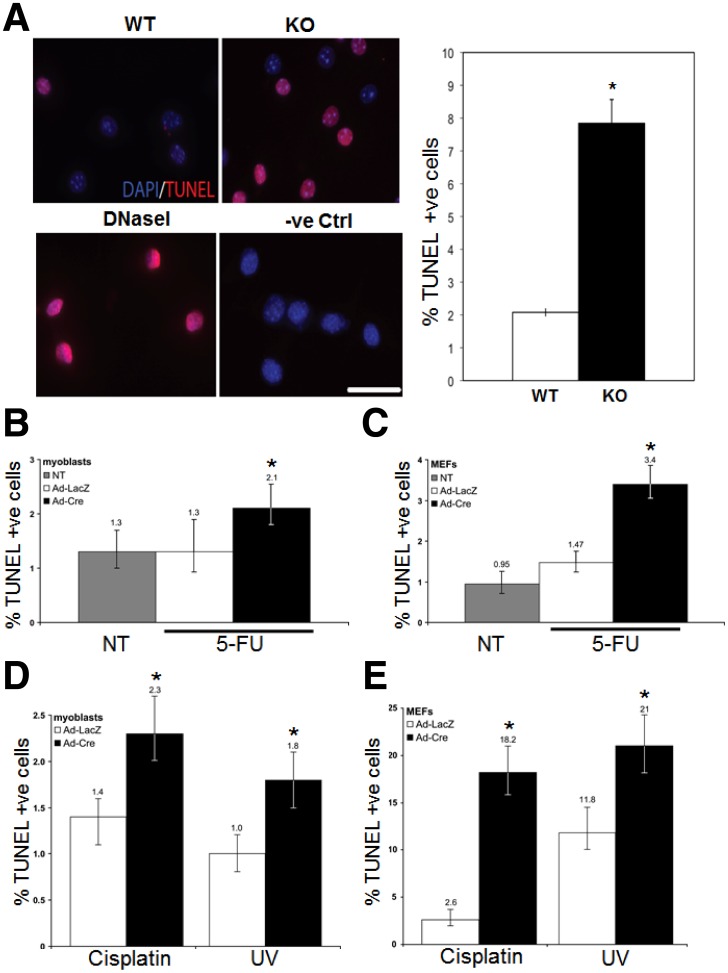
Atrx-null cells have a general sensitivity to DNA damaging agents. (A) Representative images of TUNEL positive neurons (red) isolated from wild type (WT) or *Atrx*-deficient (KO) mice following 5-FU treatment. Cells were counterstained with DAPI (blue). DNase I treatment (DNaseI) served as a positive control for TUNEL staining. Untreated cells served as a negative control (-ve Ctrl). Scale bar: 50 mM. Quantification of TUNEL positive neurons is depicted in the graph on the right. (B, C) Primary myoblasts (B) or MEFs (C) isolated from *Atrx* ^f/f^ mice were untreated (grey bar) or treated with 5-FU after infection with AdLacZ (white bar) or AdCre (black bar). Mean values are plotted with the bars corresponding to 95% confidence intervals. Asterisks denote statistically significant differences (p≤0.05). (D, E) Primary myoblasts (D) or MEFs (E) isolated from *Atrx* ^f/f^ mice were treated with cisplatin (20 mM, 24 hrs) or UV light (10 J/m^2^) after infection with AdLacZ (white bar) or AdCre (black bar). Mean values are plotted with the bars corresponding to 95% confidence intervals. Asterisks denote statistically significant differences (p≤0.05).

Cell death in neural progenitors and in our macrophage primary cell cultures can both be rescued by ablation of p53 suggesting that the integrity of the genome may be compromised in *Atrx*-null cells. Consistent with this idea, other studies have demonstrated that Atrx binds to DNA sequences that can form G4-quadruplexes, a non B-DNA structure that impinges upon cellular processes such as replication, transcription, and telomere integrity [18]. If this represents a general function of *Atrx* then other cell types lacking *Atrx* should also be sensitive to 5-FU treatment. Previously, we demonstrated that primary neurosphere cultures could be established from *Atrx* ^f/Y^:*Foxg1*-Cre mice despite a slightly compromised growth rate [20]. To address whether these cells were more sensitive to 5-FU we differentiated the cultures for 7 days then treated them with 5-FU (0.5 mM) for 18 hours before fixing and staining for DAPI and TUNEL (Fig. 4A). As a positive control for TUNEL staining, we treated one sample with DNaseI to generate fragmented DNA ends as a substrate (Fig. 4A). The proportion of TUNEL positive cells to the total number of cells (DAPI) were quantified and the Atrx-null population demonstrated a statistically significant increase in the number of TUNEL positive cells (7.8±0.7%) compared to control cultures (2.1±0.1%; n = 3; p<0.01; Fig. 4A).

To examine other cell types, we established primary myoblast and mouse embryonic fibroblast (MEFs) cultures from *Atrx* ^f/f^ mice and tested their sensitivity to 5-FU following AdCre or AdLacZ infection, similar to the macrophage experiments. As an additional control, one sample did not receive any treatment to assess death caused by virus infection. Following adenovirus infection, cells were grown for 48 hours to give adequate time to deplete the Atrx protein (Fig. S2), then treated with 5-FU (0.5 mM) for 18 hours prior to fixation and TUNEL staining. We observed a consistent but modest 1.6-fold increase in the sensitivity of *Atrx* ^null^ myoblasts to 5-FU treatment above both untreated and AdLacZ treated cells (Fig. 4B). Primary MEF cultures also showed an increased sensitivity to 5-FU treatment in the absence of *Atrx*, as we observed a 2.3-fold increase in TUNEL-positive cells (Fig. 4C). Thus, we conclude that multiple *Atrx*-null cell types are sensitive to 5-FU treatment.

To examine whether *Atrx*-null cells are sensitive to other DNA damaging agents we treated primary myoblasts and MEFs with cisplatin (20 mM, 24 hours) or UV light (10 J/m^2^) after adenovirus treatment. Significant increases in TUNEL positive cells were observed for all AdCre treated cells compared to AdLacZ treated cells (Fig. 4D, E). Taken together, these results demonstrate that cells lacking *Atrx* are more sensitive to DNA damaging agents.

## Discussion

The use of adenovirus-Cre infection to inactivate Atrx allowed us to compare macrophages that were otherwise genetically identical for their response to apoptotic stimuli. We demonstrate that Atrx-deficient cells respond normally to factors inducing the extrinsic apoptotic pathway (C2-ceramide, α-FAS, dexamethasone, and serum withdrawal) but have a heightened sensitivity to 5-FU, a pyrimidine analog known to invoke a DNA damage response and death via the p53-dependent activation of the intrinsic pathway [33]. Since this response was observed in multiple cell types and with multiple DNA damaging agents, it suggests that Atrx loss compromises genomic integrity and/or impinges on DNA repair processes in a general manner. The mechanistic basis for Atrx-dependent sensitivity to DNA damaging agents remains unknown. It is well established that Atrx is associated with heterochromatin through its localization at pericentromeric heterochromatin; interactions with heterochromatin-associated proteins including HP1α, EZH2, and MeCP2; and loading of histone H3.3 onto telomeric DNA [7], [8], [9], [10], [14]. It is possible that Atrx is required at these sites for heterochromatin formation or maintenance. In this regard, cells lacking Atrx show reduced cell growth with a telomere dysfunction phenotype in one study and mitotic catastrophe in another study [34], [35]. Other work has suggested that Atrx could function to prevent the formation of G4 quadruplex DNA structures that occur on single stranded DNA during replication or transcription [18]. Since 5-FU functions as a replication poison by depleting the available dTTP pool and through its mis-incorporation into the replicating DNA strand, one could speculate that 5-FU treatment acts as an additive effect on Atrx-null cells that are already compromised in their ability to progress through late S-phase, thus precipitating an enhanced checkpoint arrest and, ultimately, apoptosis.

Our data supports a role for Atrx in regulating genome structure and integrity and this appears to be an emerging function for this protein. HeLa cells treated with ATRX siRNA had an increased incidence of cells with bi-nucleation, intranuclear bridges and lobulated nuclei [34]. Not surprisingly, these cells had difficulty progressing through mitosis and had an increased propensity to die that was attributed to increased inter-kinetochore distance and sister chromatid cohesion. However, it is also possible that these deficits were secondary to DNA damage incurred during S-phase that was not properly repaired. Indeed, there are several examples of mitotic catastrophe and cell death arising from enhanced DNA damage, defective heterochromatin formation, or the re-establishment of heterochromatin [36], [37], [38], [39], [40]. Consistent with this idea, it has been shown that the mechanism underlying the cell lethality caused by mitotic catastrophe is through a gammaH2AX-ATM-p53-mediated apoptotic pathway [41]. Furthermore, ATRX inactivation in human tumors and mouse knockout models result in abnormal telomeres [42], [43], [44], [45]. Thus, Atrx-null cells may have impaired heterochromatin structures that invoke DNA repair pathways or, if extensive, activates p53-mediated death pathways.

While it remains to be determined if ATRX functions in re-establishing heterochromatin following replication or in DNA repair pathways, it is most similar in structure to Rad54, a Swi2/Snf2 family member that is involved in homologous recombination (HR) repair [46], [47]. A high degree of structural homology is shared between ATRX and RAD54 within their respective ATP dependent motor domains, defined by the classic 7 “helicase” motifs characteristic of the super family 2 (SF2) proteins [46]. RAD54 can resolve DNA intermediate structures (Holliday junctions) and promote the restart of DNA replication after fork stalling or collapse through its defined role in loading Rad51 onto single stranded DNA [48], [49], [50], [51]. While similar activities for ATRX have not been defined, it is prevalent at centromeres, telomeres, or G4 quadruplexes where it may be involved in targeting the HR repair machinery to facilitate replication and/or the proper organization of these DNA structures [6], [14], [35].

We demonstrated that the sensitivity to 5-FU could be rescued by removing p53, which further suggests that the DNA damage checkpoint is activated in the absence of Atrx. Consistent with this study, Seah *et al* [22] demonstrated that cell death in the developing Atrx-null cortex was through a p53-dependent apoptotic pathway. However, while cell death and brain size was rescued in the double knock-out animals, the animals still died peri-natally suggesting that Atrx may have other neuronal functions, perhaps in transcriptional regulation of specific target genes. Certainly, further work is required to determine the role of Atrx in maintaining genomic integrity and how it intersects with the p53 pathway [42], [43], [44], [45].

Somatic mutations in ATRX were identified in patients with α-thalassemia myelodysplasia (ATMDS) but recent work has shown that ATRX is also mutated in pancreatic neuroendocrine tumours and glioblastomas [28], [29], [30]. The sensitivity of multiple cell types that lack the Atrx protein to 5-FU, cisplatin, and UV light suggests that human cancers containing ATRX mutations may be similarly sensitive to these DNA damaging agents. Moreover, 5-FU and cisplatin are widely employed in the treatment of many cancers [33], [52]. Indeed, the general sensitivity of Atrx-null cells to these DNA damaging agents serves as justification for further research examining the sensitivity of ATRX cancers to such treatment regimes.

## Materials and Methods

### Primary Cell Isolation and Growth

Mice carrying the LoxP-targeted *Atrx* allele were maintained on a C57/Bl6 background and used for the isolation of primary cell cultures [20]. Primary cell cultures were established using previously established protocols for peritoneum-derived macrophages [53], myoblasts [54], neurospheres [20], and mouse embryonic fibroblasts (MEFs; [55]). Macrophage cultures were maintained in DMEM supplemented with 10% FBS and 10 ng/ml of granulocyte-macrophage colony-stimulating factor (R&D Systems). Myoblasts were cultured in Ham’s F10 medium supplemented with 20% FBS and 2.5 ng/ml bFGF. MEFs were cultured in DMEM supplemented with 10% FBS, 1% penicillin/streptomycin. Neurosphere cultures were grown in NeuroCult NSC Basal Medium supplemented with NeuroCult NSC Proliferation Supplements (StemCell Technologies) and 20 ng/ml recombinant hEGF (Invitrogen). Neurosphere differentiation was performed as described [20].

### Adenovirus Infection

The early region 1 (E1)- and E3-deleted adenovirus vectors expressing the bacteriophage P1 Cre recombinase or *E. coli* β-galactosidase from the murine cytomegalovirus immediate early enhancer/promoter have been described previously [56], [57]. These viruses were grown, purified and titered using standard techniques [58]. Due to the large size of the ATRX gene (∼7.4 kb), which cannot be easily accommodated in a traditional E1/E3-deleted Ad vector, we generated a helper-dependent Ad vector expressing ATRX. An expression cassette containing the human ATRX cDNA under regulation by the human cytomegalovirus immediate early enhancer/promoter was used to replace the β-galactosidase expression cassette in pRP2098 [59], which was subsequently amplified with the AdRP2050 helper virus [60], using standard techniques [61].

Macrophages seeded (1×10^6^ cells) on 60 mm plates were incubated with the virus at a multiplicity of infection (MOI) of 10^4^ at 37°C for 4 hours in complete media. Myoblasts or MEFs seeded at 3×10^5^ in 60 mm dishes were washed with PBS and then incubated with a 200 ml PBS solution, containing virus at an MOI of 30 (Myoblasts) or 250 (MEFs), for 1 hr at 37°C before allowing cells to recover for 48 hours in complete media. To assess the efficiency of Cre-mediated excision, genomic DNA was isolated from Ad-Cre infected cells and was PCR genotyped using primers that flank the LoxP-targeted *Atrx* allele [20]. In addition, Atrx protein was analyzed by immunoblot as described previously [7].

### Induction and Analysis of Apoptosis

Macrophage cultures infected with Ad-LacZ or Ad-Cre were allowed to recover for 24 hours prior to treatment. Following recovery, cells were exposed to LPS (20 ng/ml, 2 hours), C2-Ceramide (40 mg/ml for 24 hours), dexamethasone (100 nM for 12 hours), anti-FAS antibody (20 mg/ml for 4 hours), or 5-FU (0.5 mM for 18, 24, or 72 hours) and then TUNEL stained (Roche) to assess cell viability. Myoblasts and MEFs were exposed to 5-FU (0.5 mM) for 18 hours and then fixed and processed as above. Neurospheres were differentiated for 7 days, after which the media was removed and replaced with fresh differentiation media containing 0.5 mM 5-FU. Cells were exposed for 18 hours prior to fixation and processing by TUNEL assay.

### Cell Staining

Cells were plated on cover slips or in 4 well chamber slides and fixed with 2 or 4% PFA for one hour at room temperature. Myoblasts were cytospun (Cytospin4, Thermo Shandon) onto slides prior to fixation. Cells were permeabilized with 0.1 or 0.3% Triton-X in PBS and incubated in blocking solution (2% BSA in PBS or 20% horse serum, 0.1% fetal bovine serum, 0.03% sodium azide in PBS). Primary antibodies (rabbit anti-Atrx 1∶500; H-300, Santa-Cruz; or rat anti-mouse CD-14 1:500; BD Pharmingen) were diluted in PBS and incubated overnight at 4°C. Cells were counterstained with DAPI (1 mg/ml) for 2 minutes. Images were taken on Axio Imager M1 (Zeiss).

### Statistical Analysis

For all data sets a minimum of three biological replicates were used. For most figures, the values correspond to the mean and error bars depict the SEM. In these instances, significance was assessed using a two-sample Student’s t-test with equal variance, where p-values less than 0.05 were considered significant. For myoblast and MEF experiments (Figure 4), mean values were plotted with 95% confidence intervals shown and asterisks corresponding to significant differences (p-values <0.05).

### Study Approval

All mice used in our studies were housed and cared for according to the Canadian Council on Animal Care (CCAC) guidelines and the University of Ottawa Animal Care Committee protocols. The experimental protocols for mice (OGH/RI-23 and OGH-121) used in our studies were approved by the University of Ottawa Animal Care Committee. The standards for animal care and use conform with or exceed those defined in the Canadian Council on Animal Care’s Guide to the Care and Use of Experimental Animals, Vol. 1, 2nd edn., 1993 and the Animals for Research Act, R.S.O. 1990, c. A.22, s. 17.

## Supporting Information

Figure S1 **Cell survival of macrophages after Adenovirus treatment.** Representative images of TUNEL stained cells (green) after 5-FU treatment. Cells infected with AdCre are more sensitive to 5-FU treatment compared to AdLacZ infected cells. Co-infection with AdAtrx decreases the number of TUNEL-positive cells. Scale bar: 50 mM.
(TIF)

Figure S2 **Atrx is depleted 48 hours after AdCre treatment.** Primary MEFs were fixed and stained for Atrx (Red) two days after plating (NT, no treatment) or following infection with AdLacZ or AdCre. Nuclei are counter-stained with DAPI (Blue). Scale bar: 5 mM.
(TIF)
