# Supplementary figures and images for: Loss of Atrx Sensitizes Cells to DNA Damaging Agents through p53-Mediated Death Pathways
Source: PLoS One. 2012 Dec 17;7(12):e52167. doi: 10.1371/journal.pone.0052167 (PMC3524112; doi:10.1371/journal.pone.0052167)

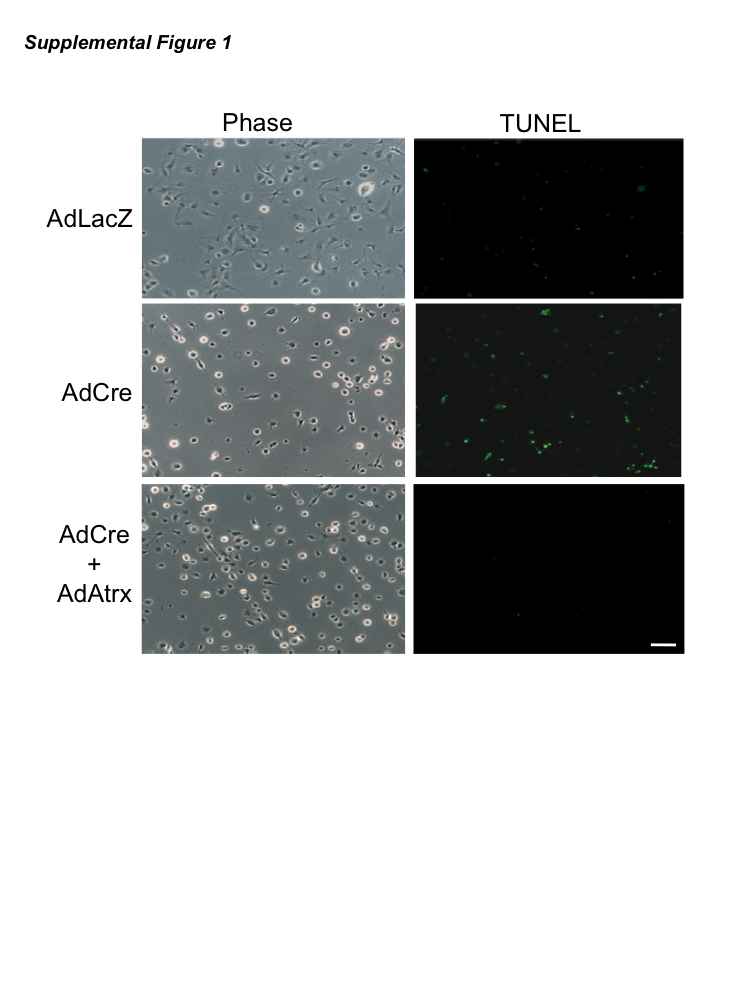

Supplement: Figure S1 — Cell survival of macrophages after Adenovirus treatment. Representative images of TUNEL stained cells (green) after 5-FU treatment. Cells infected with AdCre are more sensitive to 5-FU treatment compared to AdLacZ infected cells. Co-infection with AdAtrx decreases the number of TUNEL-positive cells. Scale bar: 50 mM. (TIF) [file pone.0052167.s001.tif]

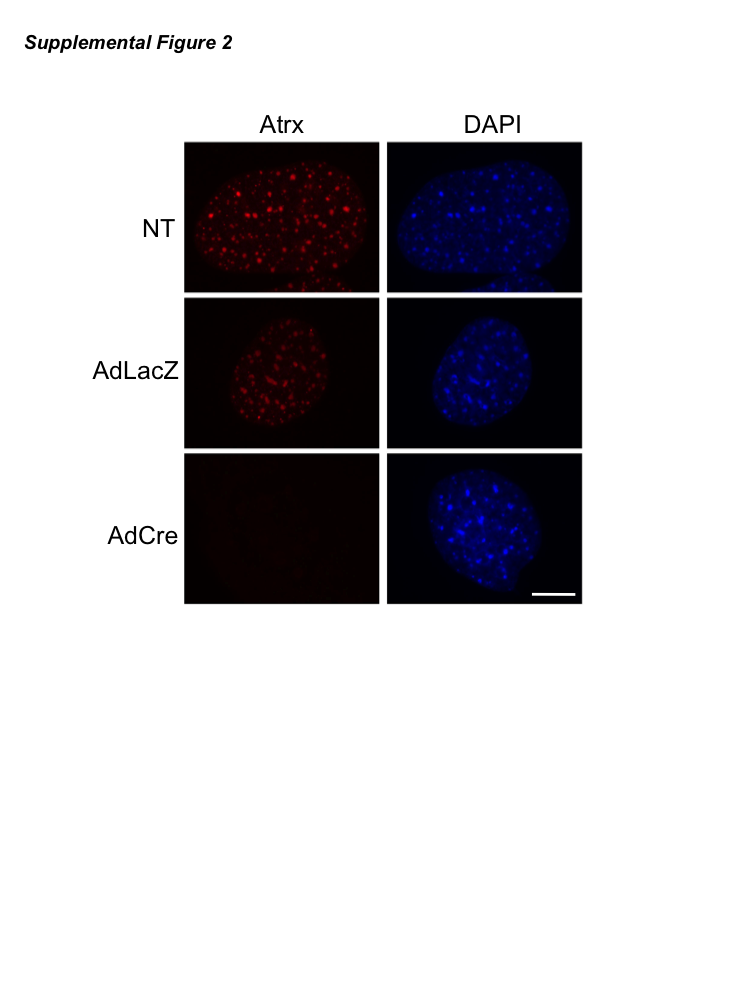

Supplement: Figure S2 — Atrx is depleted 48 hours after AdCre treatment. Primary MEFs were fixed and stained for Atrx (Red) two days after plating (NT, no treatment) or following infection with AdLacZ or AdCre. Nuclei are counter-stained with DAPI (Blue). Scale bar: 5 mM. (TIF) [file pone.0052167.s002.tif]
